# The Wilms’ tumor gene-1 is a prognostic factor in myelodysplastic syndrome: a meta analysis

**DOI:** 10.18632/oncotarget.23671

**Published:** 2017-12-27

**Authors:** Yanan Jiang, Lin Liu, Jinhuan Wang, Zeng Cao, Zhigang Zhao

**Affiliations:** ^1^ Tianjin Medical University Cancer Institute and Hospital, National Clinical Research Center for Cancer, Key Laboratory of Cancer Prevention and Therapy, Tianjin’s Clinical Research Center for Cancer, Tianjin 300060, China; ^2^ Department of Oncology, Second Hospital of Tianjin Medical University, Institute of Urology, Tianjin 300060, China

**Keywords:** myelodysplastic syndrome (MDS), wilms tumor-1 (WT1), prognostic factor, meta-analysis, survival

## Abstract

Previous studies have suggested that Wilms’ tumor gene-1 (WT1) may be related to a decrease in both relapse-free survival (RFS) and overall survival (OS) for patients with myelodysplastic syndrome (MDS). Therefore, we conducted a meta-analysis on the utility of WT1 as a prognostic indicator of MDS. Published reports were searched in the following databases: Cochrane Library, PubMed, Embase, and Web of Science. The meta-analysis was conducted using the Cochrane Collaboration RevMan 5.2 software. Six publications with 450 total patients met the inclusion criteria and were subjected to further examination. The results showed a reduction in both overall survival (OS) and leukemia-free survival (LFS) with increasing WT1 expression levels: 1-year OS (odds ratio, OR = 0.16; 95% CI = 0.08–0.34, *P* < 0.001), 3-year OS (OR = 0.21; 95% CI = 0.09–0.47, *P* < 0.001), 5-year OS (OR = 0.24; 95% CI = 0.06–0.92, *P* = 0.04), 1-year LFS (OR = 0.06; 95% CI = 0.02–0.18; *P* < 0.001), 3-year LFS (OR = 0.20; 95% CI = 0.09–0.46; *P* < 0.001), and 5-year LFS (OR = 0.12; 95% CI = 0.04–0.38; *P* < 0.001). In terms of patients receiving hematopoietic stem cell transplantation, the cumulative incidence of relapse (CIR) was higher in the WT1 over-expression group than in the low-expression group: 1-year CIR (OR = 13.69; 95% CI = 2.99–62.62; *P* < 0.001), 3-year CIR (OR = 6.52; 95% CI = 2.31–18.40, *P* < 0.001). In conclusion, WT1 over-expression is a prognostic factor for MDS.

## INTRODUCTION

Myelodysplastic syndrome (MDS) is a group of hematological diseases that present as cytopenia, dysplastic bone marrow (BM), or a pre-leukemic condition can cause patients to progress to acute myeloid leukemia (AML) [1–3]. Relying on conventional cytogenetics, cytomorphology, and peripheral blood parameters, the International Prognostic Scoring System (IPSS) and other models [4–6] have been successfully used for accurate diagnoses and treatment of various conditions, with a recent revision of the IPSS (IPSS-R) further improving prognostic risk stratification and optimal treatment selection. Frequently, MDS cannot be cured by chemotherapy alone, thereby requiring hematopoietic stem cell transplantation (HSCT). However, HSCT is not able to prevent MDS relapse in all patients. At present, molecular markers have been extensively used to monitor minimal residual disease (MRD), and thereby evaluate the potential for relapse in hematological malignancies. Although specific markers for MDS are not always available, Wilms’ tumor gene-1 (WT1) has been recommended as a universal marker, but has not yet been established clinically.

WT1, located in chromosome 11p13, was first cloned gene that was a suppressor in Wilms’ tumor, which encodes a zinc finger transcription factor [7]. WT1 expression is found in a small number of normal tissues [8], such as kidney, stromal cells of the uterus, testis, ovaries, and myometrium [9]. WT1 mRNA is overexpressed in various solid cancers, as well as hematologic malignancies, including AML [6], acute lymphocytic leukemia (ALL) [5], chronic myeloid leukemia (CML), and MDS [10–11]. Overexpression of WT1 is found in 89–100% of patients with AML and MDS [12–15]. AML patients with high WT1 expression levels are reported to have a relatively poor prognosis [16–18], with several studies [19–20] suggesting that WT1 expression may be related to MDS prognosis.

In this study, 6 publications with a total of 450 patients with MDS were pooled in the meta-analysis. The aim of this study was to use a meta-analysis to determine any association between WT1 expression status and overall survival (OS), leukemia-free survival (LFS), and cumulative incidence of relapse (CIR) for patients with MDS.

## RESULTS

### Study characteristics

**Figure 1:**
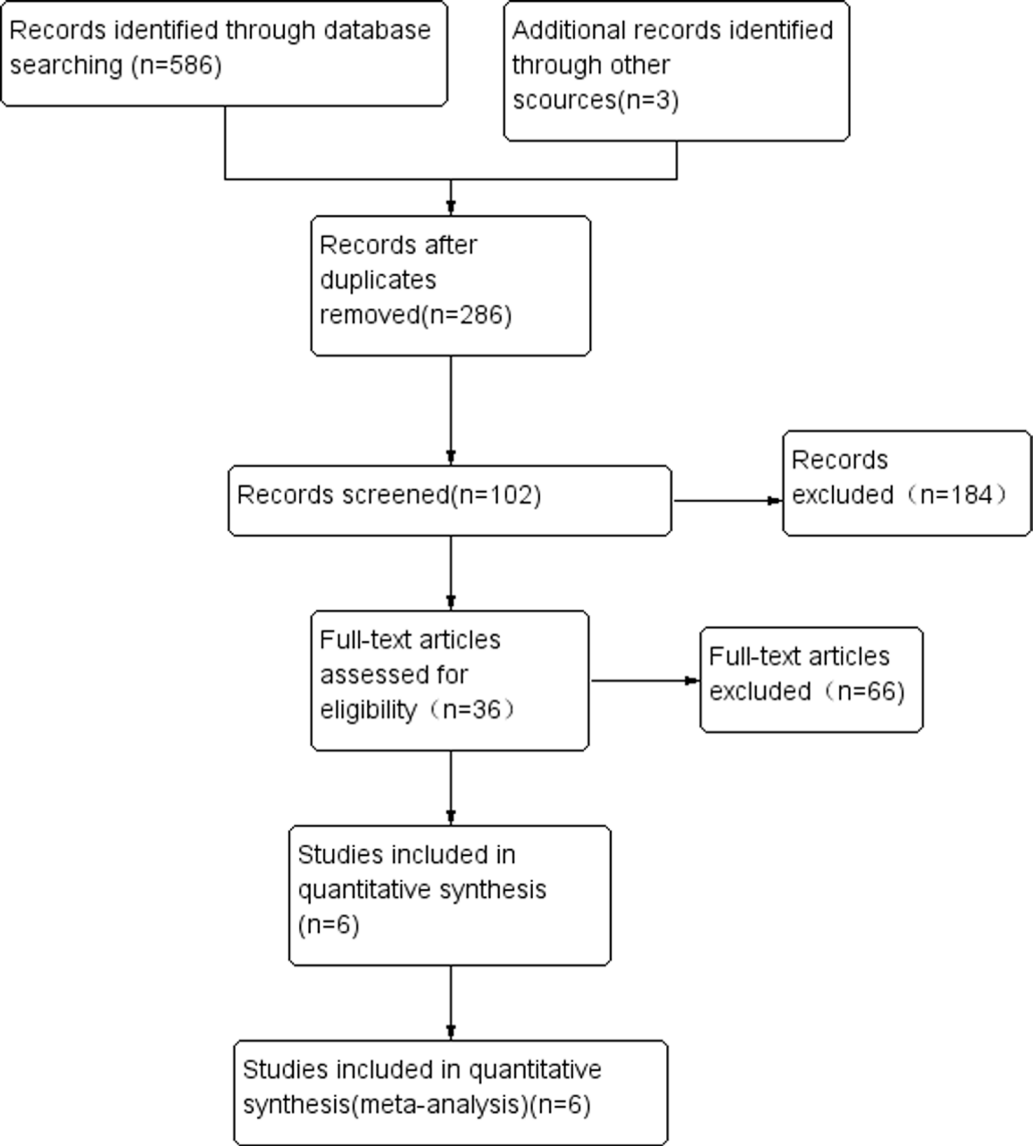
Study flow diagram of included studies

The flow chart in Figure 1 summarizes the literature review process utilized in this meta-analysis. Six publications were included in the analysis of this study. Of these publications, 2 papers examined the prognostic utility of WT1 expression after HSCT for MDS. One publication analyzed only elderly patients. Characteristics of the included studies are shown in Table 1.

**Table 1:** Main characteristics of studies included in the meta-analysis

Author	Year	Sample size	Male/female	age	Results
Sumiko [27]	2016	82	50/32	64.6	OS LFS
Joji [28]	2017	42	23/19	73	OS
Paola [20]	2015	86	49/37	NA	OS LFS
Hideto [26]	2010	80	53/27	70	OS LFS
Jae-Ho [37]	2015	82	53/29	49	OS CIR
Mo XD [36]	2016	78	48/30	38	CIR

Abbreviations: NA: not available, OS: overall survival, LFS: leukemia-free survival, CIR: cumulative incidence of relapse.

### Relationship between WT1 and overall survival for MDS

**Figure 2:**
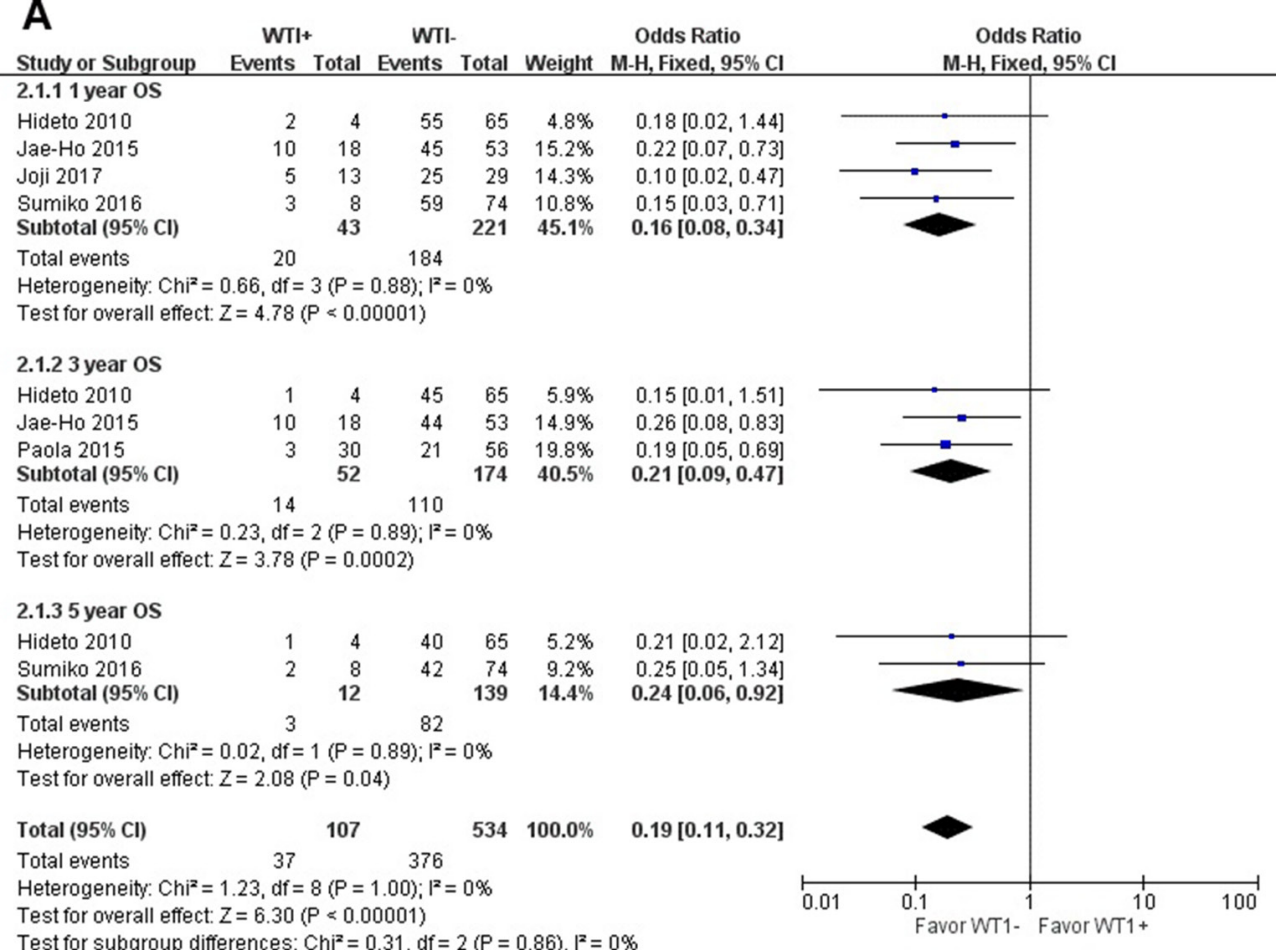

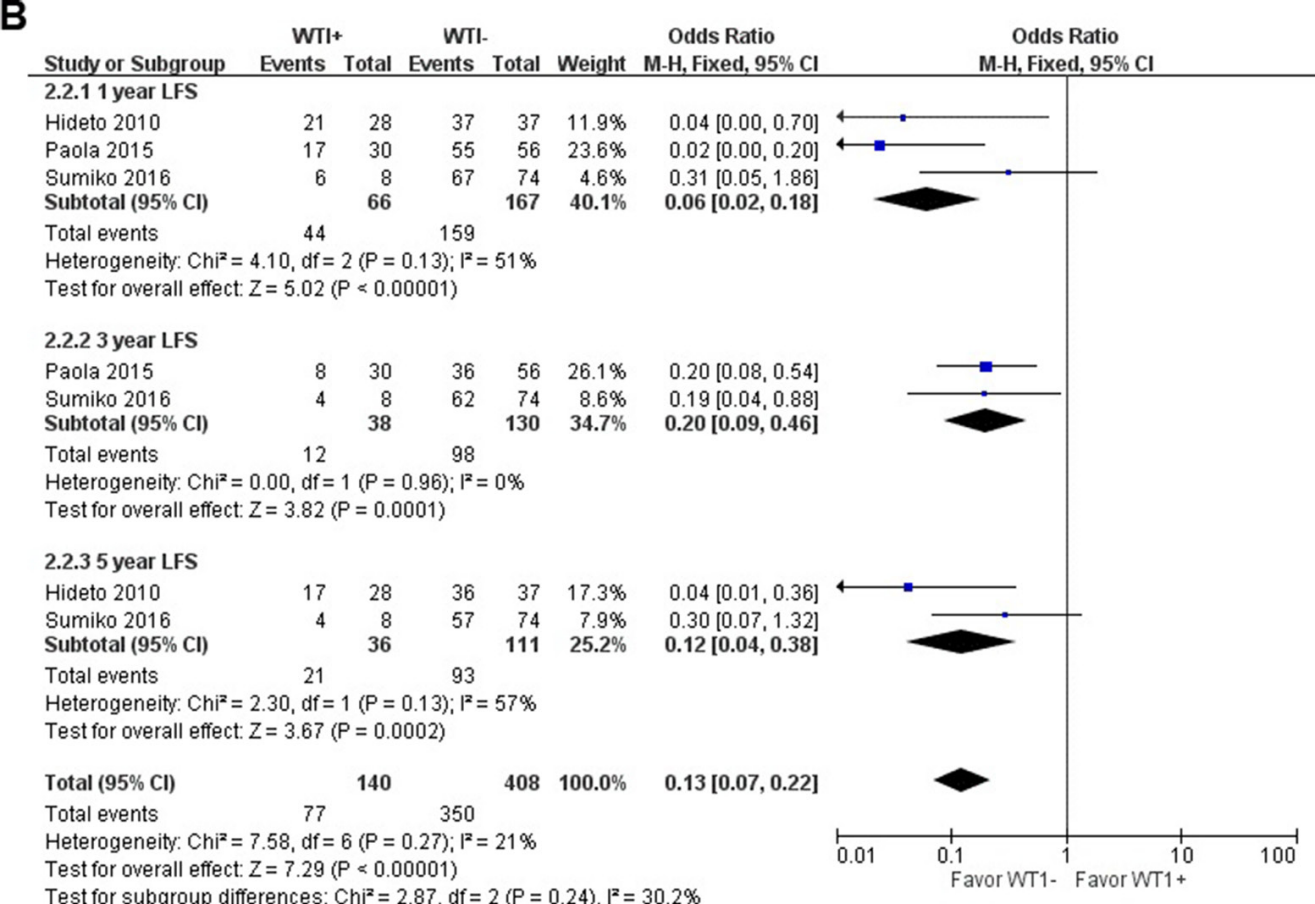

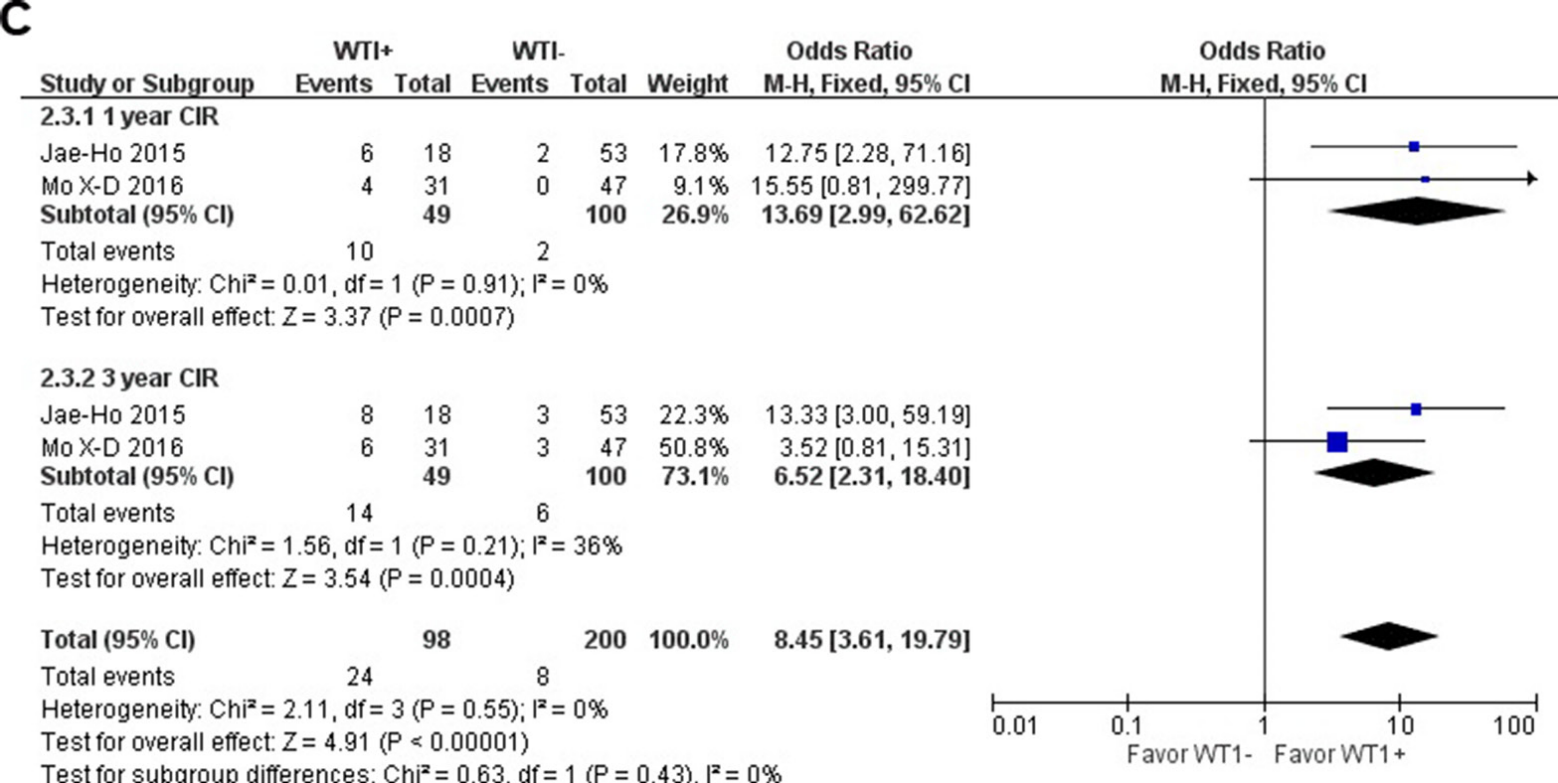
Meta-analysis of the association between WT1 and OS (**A**), LFS (**B**), CIR (**C**) of MDS, respectively.

When all eligible studies were pooled into one dataset for the meta-analysis, we found that WT1 was significantly associated with OS in MDS. Further subgroup analysis of OS at 1, 3, and 5 years showed that when compared to the WT1 over-expression group, outcomes in the WT1 low-expression group were significantly better (1-year OS: odds ratio, OR = 0.16; 95% confidence interval (CI) = 0.08–0.34; *P* < 0.001; 3-year OS: OR = 0.21; 95% CI = 0.09–0.47; *P* < 0.001; and 5-year OS: OR = 0.24; 95% CI = 0.06–0.92; *P* = 0.04). These results are shown in Figure 2A.

### Relationship between WT1 and leukemia-free survival for MDS

When we assessed LFS in patients with MDS, significant differences were observed between patients with low- and over-expression of WT1 (OR = 0.13; 95% CI = 0.07–0.22; *P* < 0.001). All LFS time periods examined, 1-year, 3-year, and 5-year LFS, showed better in outcomes in the WT1 low-expression patients than over-expression patients (1-year LFS: OR = 0.06; 95% CI = 0.02–0.18; *P* < 0.001; 3-year LFS: OR = 0.20; 95% CI = 0.09–0.46; *P* < 0.001; 5-year LFS: OR = 0.12; 95% CI = 0.04–0.38; *P* < 0.001). The results of LFS are shown in Figure 2B.

### Relationship between WT1 and cumulative incidence of relapse after HSCT for MDS

As for CIR for MDS patients after HSCT, the CIR was higher in the WT1 over-expression group than in the low-expression group. The results are as follows: 1-year CIR (OR = 13.69; 95% CI = 2.99–62.62; *P* < 0.001) and 3-year CIR (OR = 6.52; 95% CI = 2.31–18.40; *P* < 0.001). The results of CIR are shown in Figure 2C.

### Sensitivity analyses and publication bias

**Figure 3:**
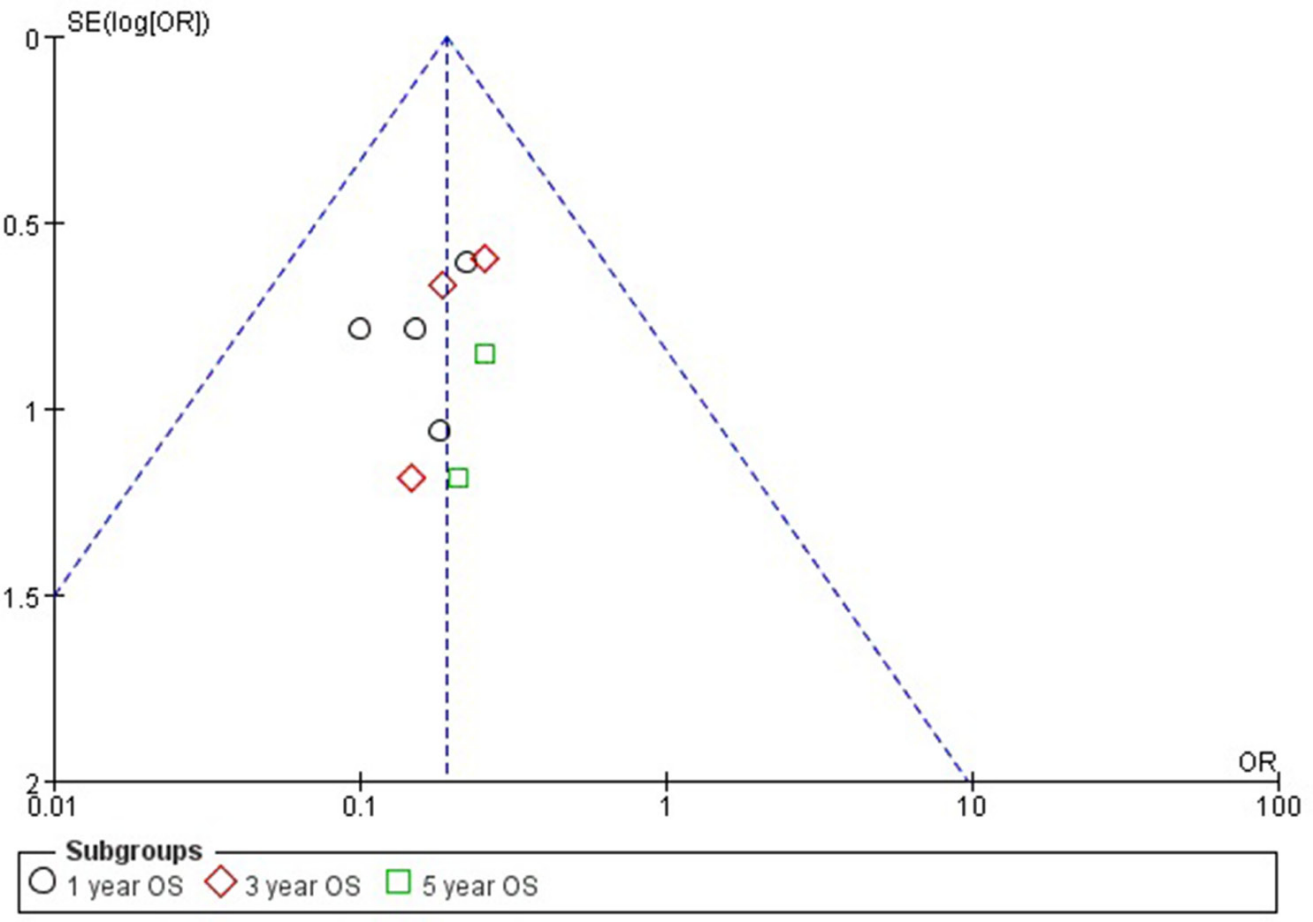
Funnel plot of the association between WT1 expression and OS of MDS

The result of the shape of the OS funnel plot appeared symmetrical, as can be seen in Figure 3. This symmetry in the OS funnel plot suggests that there was no obvious publication bias. However, due to the low number of publications included in this meta-analysis, we did not analyze bias in LFS or CIR.

## DISCUSSION

WT1 is over-expressed in many malignant hematologic diseases [21], with this gene expression already being used in the monitoring of AML patients and is also being investigated for use in immunotherapy [10]. A detailed understanding of the role of WT1 in malignant hematologic diseases may improve and consolidate its utilization in the clinical setting. However, the relationship between WT1 mRNA expression status and the prognosis of MDS has not been comprehensively investigated. We analyzed the relationship between WT1 expression status and OS, LFS, and CIR for patients with MDS, which showed that the overexpression of WT1 may be associated with a poor prognosis.

Previous studies have shown that WT1 expression levels increase as the MDS disease stage progresses, in accordance with IPSS [22, 23], the World Health Organization’s classification-based Prognostic Scoring System (WPSS) [24], and the revised IPSS (IPSS-R) [25]. Of the studies included in this meta-analysis, two reported that WT1 mRNA expression showed a higher frequency in the higher-risk group, as classified by either WPSS or IPSS-R categories. Tamura *et al*. [26] examined the relationship between WT1 over-expression and the different risk groups, as classified by WPSS. High WT1 expression was observed in 9 of 25 patients (36%), 4 of 9 (44.4%), 2 of 5 (40%), 8 of 11 (72.7%), 12 of 13 (92.3%), 15 of 16 (93.8%), and 1 of 1 (100%) in the RA, RCMD, RARS, RAEB-1, RAEB-2, AML-MDS, and 5q- categories, respectively. Kobayashi *et al*. found that elevated WT1 expression levels were also more prevalent in the high-risk group, as classified by IPSS-R categories [27]. In the different risk groups of MDS, high WT1 expression levels were 2 of 8 patients (25%), 10 of 29 (34.5%), 12 of 21 (57.1%), 9 of 11 (81.8%), and 11 of 13 (84.6%) in the very-low, low, intermediate, high, and very-high risk categories, respectively. Therefore, from these studies we can conclude that WT1 mRNA expression levels increase in accordance with the aggressiveness of MDS disease subtypes, in direct relation to patient prognosis.

Nagasaki *et al*. [28] studied the relationship between progression-free survival (PFS) and WT1 expression status. In this study, the 1-year PFS was 13 of 29 patients (44.8%) and 9 of 13 (69.2%) in the high- and low-expression groups, respectively. Median survival was reported in 4 of the analyzed publications in our meta-analysis. Kobayashi *et al*. [27] showed that the median OS of patients with WT1 mRNA expression levels of 500, 50000, 100000, 150000 copies were greater than 60.0, 45.0, 17.5 and 12.0 months, respectively. In the report of Nagasaki *et al*. [28], median OS of the low- and high-expression groups of WT1 were 54 and 10 months, respectively. Similarly, Minetto *et al*. [20] reported a median OS of 35 and 21 months, for the low- and high-expression groups, respectively. Furthermore, Tamura *et al*. [26] found a median OS for the low-, intermediate-, and high-expression groups of WT1 to be 96, 60, and 12 months, respectively. Four studies examined OS, while 3 studies investigated LFS in this meta-analysis. These results showed a decrease in both PFS and OS as the WT1 mRNA expression level increased, thereby showing WT1 expression as a prognostic factor for MDS patients.

For patients with MDS, allogenic HSCT is the only known potentially curative treatment; however, a large portion of these patients still have relapsed. Previous studies have shown the probability of CIR to be 19–51%, even with the application of conditioning regimens using myeloablation [29]. Subsequent therapeutic options are limited for patients with relapse after HSCT, and the survival rates of these patients are less than 20% [30, 31]. Therefore, it is crucial to predict any potential relapse to administer preemptive interventions. The presence of MRD after HSCT can indicate and detect impending relapse. Many genetic mutations in MDS, such as TP53, RUNX1, and ETV6, are suboptimal biomarkers for detecting relapse of MDS, but none have been applied for the detection of MRD in the HSCT setting [32–35]. The WT1 expression level is considered a universal marker for MRD in MDS [12]. In this meta-analysis, 2 studies detected relapse after allogenic HSCT [36, 37]. For MDS patients treated with HSCT, the 1- and 2-year CIR were significantly higher for patients with WT1 over-expression. Therefore, MRD monitoring using WT1 can potentially identify MDS patients who may have a higher risk of relapse after HSCT. An important point needs to be highlighted that the WT1 expression levels in these two studies were detected after transplantation. For MDS patients with HSCT, the WT1 expression levels and prognostic implications differed between pre- and post-HSCT status. Compared with WT1 expression levels measured post-HSCT, which is useful for relapse prediction in MDS, the role of the pre-HSCT WT1 expression status is controversial. According to Yoon *et al*., the role of pre-HSCT WT1 expression showed weak correlations to both BM blast counts and IPSS scores at the time of HSCT [37]. A contradictory conclusion by Woehlecke *et al*., stated that for childhood MDS patients, high levels of pre-transplantation WT1 expression were associated with a higher CIR, lower event free survival (EFS), and OS [38]. Therefore, more studies are needed to identify whether the level of pre-HSCT WT1 expression influences the prognosis of MDS patients treated with HSCT.

Our study had multiple limitations.First, due to the limited number of patients included in our study, we could not perform a MDS subgroup analysis for multiple WT1 expression levels, other than binary high/low levels. Second, the sample size of this meta-analysis was relatively small, with the significance of the WT1 gene for HSCT patients remains to be further explored. Third, the treatment regimens for study patients were heterogeneous, including immuno-chemotherapy and HSCT, which may introduce some study bias.

As stated in Ueda *et al*., the relatively rapid quantitation of WT1 mRNA, a possible novel marker to complement the current IPSS, WPSS, and IPSS-R criteria, is considered to be a useful test to determine the prognosis of MDS and has potential for clinical application [21]. From our meta-analysis, we can conclude that WT1 is a significant prognostic factor of MDS. In addition, studies elucidating the immune pathology of MDS are warranted. Introduction of an appropriate immune response to WT1 molecules may be beneficial in optimizing prognosis [11].

## MATERIALS AND METHODS

### Publication search

Published report were searched in the following databases: Cochrane Library, PubMed, Embase, and Web of Science. The meta-analysis was conducted using the Cochrane Collaboration RevMan 5.2 software (The Cochrane Collaboration, London, United Kingdom), with the following keywords: “myelodysplastic syndrome or MDS” and “Wilms tumor gene 1 or WT1”. We defined the literature type as clinical trial, or prospective or retrospective cohort studies. Publication language was not restricted in this search.

### Study selection

Six studies were pooled into this meta-analysis. Studies were included if: (1) study design was prospective or retrospective cohort study; (2) the exposure of interest was WT1; (3) the studies reported relative risks (RRs), or ORs with corresponding 95% CIs; (4) the study outcome was OS, LFS, or CIR, or regarding different subtypes and other outcomes that can represent prognosis. If the same cohort was used in more than one publication, we included the publication that reported the results in greater detail. However, if the details of the multiple studies were similar, the one with the largest number of cases was analyzed. Data published as only abstracts were excluded. Case reports and review articles were also excluded.

### Review strategy and procedure

Initially, the search returned 586 publications from scientific, peer-reviewed journals. Once duplicates were removed, 286 publications were included in the preliminary set. One hundred eighty-four studies that did not have clinical data regarding the prognosis of MDS were excluded through inspection of the titles and abstracts, and studies published in abstract form only were also excluded. After a full-article review, 66 studies were excluded due to insufficient data for quantitative analysis, or because the studies were either case reports and review articles, leaving 36 publications in the analysis. Another 30 of 36 publications were excluded because of duplicated data, or lack of sufficient data about OS, LFS, or CIR. Finally, a total of 6 publications were included in the meta-analysis.

### Data extraction

The following data were extracted from each eligible study by two investigators: the first author, year, study design, number of patients included in analysis, sex, age, and ORs with corresponding 95% CIs for OS, LFS, and CIR.

### Statistical analysis

The strength of the association between WT1 expression and OS, LFS, and CIR for MDS was evaluated by calculating the ORs and 95% CIs. To assess the significance of the ORs, we conducted the *Z*-test, with statistical significance achieved when the *P* value was less than 0.05. Moreover, the chi-squared-based *Q* and *I*^2^ tests were performed to evaluate the inter-study heterogeneity, with *P* < 0.1 being defined as statistical significant. The random-effects model was used to calculated the ORs if significant heterogeneity existed; otherwise, the fixed-effects model was applied. Publication bias was assessed by the asymmetry of funnel plots. We conducted all analyses using the Review Manager 5.1 software (Nordic Cochrane Center, Copenhagen, Denmark).
